# Expression and processing of mature human frataxin after gene therapy in mice

**DOI:** 10.21203/rs.3.rs-3788652/v1

**Published:** 2023-12-28

**Authors:** Teerapat Rojsajjakul, Nithya Selvan, Bishnu De, Jonathan B. Rosenberg, Stephen M. Kaminsky, Dolan Sondhi, Peter Janki, Ronald G. Crystal, Clementina Mesaros, Richie Khanna, Ian A. Blair

**Affiliations:** University of Pennsylvania Philadelphia; LEXEO Therapeutics, Inc; Weill Cornell College of Medicine; Weill Cornell College of Medicine; Weill Cornell College of Medicine; Weill Cornell College of Medicine; LEXEO Therapeutics, Inc; Weill Cornell College of Medicine; University of Pennsylvania Philadelphia; LEXEO Therapeutics, Inc; University of Pennsylvania Philadelphia

## Abstract

Friedreich’s ataxia is a degenerative and progressive multisystem disorder caused by mutations in the highly conserved frataxin (FXN) gene that results in FXN protein deficiency and mitochondrial dysfunction. While gene therapy approaches are promising, consistent induction of therapeutic FXN protein expression that is sub-toxic has proven challenging, and numerous therapeutic approaches are being tested in animal models. FXN (hFXN in humans, mFXN in mice) is proteolytically modified in mitochondria to produce mature FXN. However, unlike endogenous hFXN, endogenous mFXN is further processed into N-terminally truncated, extra-mitochondrial mFXN forms of unknown function. This study assessed mature exogenous hFXN expression levels in the heart and liver of C57Bl/6 mice 7–10 months after intravenous administration of a recombinant adeno-associated virus encoding hFXN (AAVrh.10hFXN) and examined the potential for hFXN truncation in mice. AAVrh.10hFXN induced dose-dependent expression of hFXN in the heart and liver. Interestingly, hFXN was processed into truncated forms, but found at lower levels than mature hFXN. However, the truncations were at different positions than mFXN. AAVrh.10hFXN induced mature hFXN expression in mouse heart and liver at levels that approximated endogenous mFXN levels. These results demonstrate that AAVrh.10hFXN may induce expression of therapeutic levels of mature hFXN in mice.

## INTRODUCTION

Friedreich’s ataxia (FRDA) is the most common inherited ataxia, affecting approximately 1 in 50,000 of the population in the US, mainly Caucasian^1^. Symptoms include dysarthria (slurred speech), spasticity, scoliosis, diabetes, processive ataxia, and cardiomyopathy^1–6^. Prognosis is poor, with patients slowly progressing to wheelchair dependency (usually in their late teens or early twenties^2^) and the majority dying from heart disease between the ages of 16 and 41^7,8^. While there are currently no curative treatments for the cardiac manifestations of FRDA, numerous therapeutic approaches are being tested in pre-clinical models^9–11^ As FRDA is a genetic disease caused by an autosomal recessive mutation in the frataxin gene (FXN) that encodes the highly conserved frataxin protein (*FXN*), genetic approaches for treatment are under investigation^12–14^. Mutations causal for FRDA classically consist of a trinucleotide (GAA) repeat expansion within the first *FXN* intron, which results in DNA triplex formation, epigenetic silencing, transcription inhibition, and consequently, reduced FXN protein production^15–17^. While the intracellular role of FXN has not been clearly defined^18,19^, FXN deficiency compromises iron-sulfur (Fe-S) cluster formation, leading to mitochondrial iron overload, mitochondrial dysfunction, and oxidative stress^20^ that can culminate in the neuro- and cardio-degeneration characteristics of FRDA^21–24^.

Gene replacement (e.g., gene therapy) represents a promising approach to treating FRDA^10,11^. One of the pivotal challenges critical to the success of gene therapy is to induce sufficient FXN protein expression to achieve therapeutic efficacy while limiting the toxicity induced by its overexpression, which can result in impaired formation of Fe-S clusters and cellular/mitochondrial function, and eventually lead to cell death^17^. Previous gene therapy studies conducted in mouse models have shown human FXN (hFXN) expression at levels associated with cardiotoxicity, hepatotoxicity, and therefore, reduced efficacy^25,26^. Thus, gene replacement approaches for FRDA continue to require detailed study in animal models to optimize efficacy and minimize toxicity in human studies.

**Figure 1:**
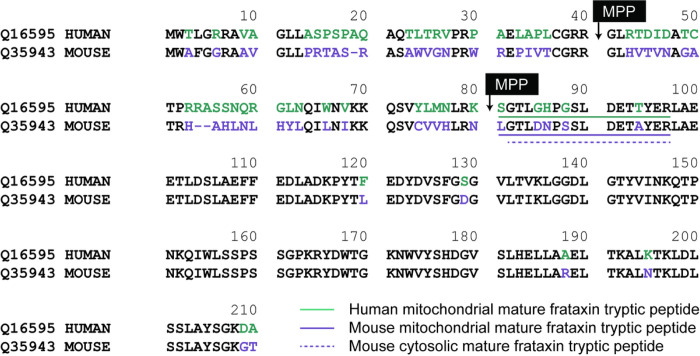
Alignment of mature hFXN (Uniprot Q16595) and mFXN (Uniprot Q35943). Amino acids in green are specific to hFXN while those in purple are specific to mFXN. Translocation of full-length FXN to the mitochondria followed by two sequential cleavages by MPP results in the formation of mature hFXN and mFXN as 130 amino acid proteins. Quantification of mature hFXN is based on the S^81^GTLGHPGSLDETTYER^97^ tryptic peptide (N), while quantification of mature mFXN is based on the L^78^GTLDNPSSLDETAYER^94^ tryptic peptide (N). Extra-mitochondrial mature mFXN is formed through an unknown mechanism and is detected as the truncated N-1 tryptic peptide G^79^TLDNPSSLDETAYER^94^. Other proteoforms are detected by further truncations on the N-terminal tryptic peptide as N-2, N-3, N-4, N-5, and N-6. hFXN = human frataxin; mFXN = mouse frataxin; MPP = mitochondrial processing peptidase.

While FXN is highly conserved, species-specific processing results in diverse populations of mature FXN variants. Full-length hFXN (1–210) is expressed in the cytosol of nucleated cells and translocates to mitochondria for two-step removal of the mitochondrial targeting sequence by mitochondrial processing peptidase (MPP) ^27^. This results in production of a 130-amino acid, mitochondrial mature hFXN protein (81–210, Fig. 1) ^28,29^. In addition to this hFXN form, an alternatively spliced, N-terminally acetylated (methionine-76) extra-mitochondrial, 135-amino acid hFXN form was discovered in human erythrocytes and termed hFXN-E^24,30^. Mitochondrial mature hFXN and extra-mitochondrial mature hFXN-E proteins are not secreted into systemic circulation but can be quantified in whole blood of healthy control subjects and FRDA patients^16,17^. A high-resolution mass spectrometry (HRMS)-based method was developed for the analysis of hFXN to characterize mitochondrial mature mouse FXN (mFXN, 78–207; Fig. 1), which is thought to arise by the same MPP-dependent mechanism as mature hFXN^31,32^. However, it was found that mice also form a major extra-mitochondrial form of mature mFXN in which the N-terminal leucine residue is lost to generate a cytosolic 129-amino acid truncated protein with an N-terminal tryptic peptide at the amino terminus (G^79^TLDNPSSLDETAYER^94^; Fig. 1) ^31^. Other mFXN truncations include a major N-3 truncation that gives rise to a mature mFXN proteoform in the liver with an N-terminal L^81^DNPSSLDETAYER^94^ peptide (Fig. 1) ^31^. Interestingly, hFXN does not undergo such post-translational processing to form truncated extra-mitochondrial forms in humans^28,29^, and mice cannot produce the alternatively spliced extra-mitochondrial FXN-E form observed in humans and non-human primates as mFXN lacks the corresponding methionine-76 residue of hFXN^31^. Thus, while mitochondrial mature hFXN and mFXN arise by similar mechanisms, species-specific variants exist and must be considered in pre-clinical studies on hFXN gene therapy.

The primary objective of this pre-clinical study was to determine whether intravenous (IV) gene therapy with recombinant adeno-associated virus rhesus serotype 10 encoding hFXN (AAVrh.10hFXN) could drive expression of mature hFXN in wild-type (WT) mice at levels that approximate endogenous mature mFXN^31^ using a two-dimensional nano-ultra-high performance liquid chromatography-parallel reaction monitoring (2D-nano-UHPLC-PRM/HRMS) method^31^ to quantify both endogenous mature mFXN and exogenous mature hFXN in heart and liver tissues. The secondary objective of this study was to determine whether hFXN expressed in mouse tissues undergoes post-translational truncation—a process that does not occur in human tissues^31^.

## METHODS

### Reagents and supplies.

All reagents and solvents were liquid chromatography (LC)/mass spectrometry (MS)-grade quality unless otherwise noted. [^13^C_6_]-leucine was obtained from Cambridge Isotope Laboratories (Andover, MA). Anti-FXN recombinant rabbit monoclonal antibody (mAb) EPR21840 (cat. # ab219414) was from Abcam (Cambridge, UK). Ethylenediaminetetraacetic acid (EDTA)-free protease inhibitor cocktail, DL-dithiothreitol (DTT), EDTA-free Easypack protease inhibitor cocktail tablets, and dimethyl pimelimidate dihydrochloride (DMP) were purchased from MilliporeSigma (Billerica, MA). LC-grade water and acetonitrile were obtained from Burdick and Jackson (Muskegon, MI). Protein G Dynabeads for immunoprecipitation (IP) and radioimmunoprecipitation assay (RIPA) lysis buffer with EDTA were from ThermoFisher Scientific (Waltham, MA). LC/MS grade water and Optima LC/MS grade solvents were from Fisher Scientific (Pittsburgh, PA).

### Study animals.

The study was conducted in accordance with ARRIVE guidelines. C57Bl/6 mice were received from Taconic Biosciences (Germantown, NY) and were weighed and identified by ear punch upon receipt. Animals were housed by sex and treatment group in normal vivarium cages, with up to four mice per cage. Temperature and relative humidity were maintained at 21–23°C and 45–55%, respectively, and were monitored by Watchdog Environmental Monitors (VERTIV; Lincoln, NE). Animals were exposed to a 12:12 h light:dark cycle that alternated at 6 am and 6 pm. Water was supplied through an automated watering system, and the water pH was maintained at 2.5–2.8 (monitored weekly). Food was provided *ad libitum* (PicoLab^®^ Rodent Diet 20; cat. # 5053; Lab Supply; Northlake, TX).

### Dosing and sampling.

For the purposes of 2D-nano-UHPLC-PRM/HRMS analysis, eight-week-old (± 1 week) WT C57Bl/6 mice weighing between 18–30 g were IV-administered vehicle [control article/formulation buffer (phosphate-buffered saline [PBS], pH 7.4; 1.06 mM KH_2_PO_4_, 155.17 mL NaCl, 2.97 mM Na_2_PO4 7H_2_O)] or a low [1.8e12 genome copies (gc)/kg)], mid (5.7e12 gc/kg), or high (1.8e13 gc/kg) dose of AAVrh.10hFXN. Vector titration was performed by quantitative polymerase chain reaction (data not shown). Administration was performed in a volume of 0.1 mL by IV injection into the lateral veins of the tail. The control group (n = 5) comprised three males and two females, the low-dose and mid-dose groups (n = 6 each) comprised three males and three females, and the high-dose group (n = 8) comprised three males and five females. Animals were excluded from the study when the IV injection leaked subcutaneously or externally, or mice did not survive for 48-h post injection, or dermatitis and/or any signs of severe stress and pain were noted 48 h after injection. Mice were anesthetized by CO_2_ inhalation until surgical plane, then euthanized by terminal cardiocentesis 7–10 months after dosing. Heart (septum) and liver were collected, weighed, and snap-frozen on dry ice and stored at −80°C (± 10°C) until further processing.

### Internal standard.

**Figure 2:**
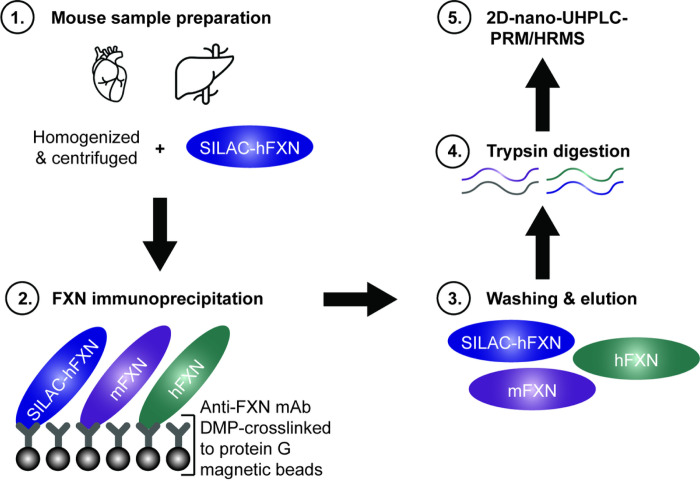
Workflow for quantification of mature hFXN and mFXN. Covalent linking of a rabbit mAb to protein G beads facilitated efficient recovery of mFXN and hFXN proteoforms from tissues for 2D-nano-UHPLC-PRM/HRMS analysis, as it allowed the beads to be washed to remove matrix contaminants. 2D-nano-UHPLC-PRM/HRMS = two-dimensional nano-ultra-high performance liquid chromatography-parallel reaction monitoring high-resolution mass spectrometry; DMP = dimethyl pimelimidate dihydrochloride; FXN = frataxin; hFXN = human frataxin; mAb = monoclonal antibody; mFXN = mouse frataxin; SILAC = stable isotope labelling by amino acids in cell culture.

It was possible to determine whether truncation of the FXN proteins had occurred *in vivo* in the mice or during sample processing by adding an internal standard (stable isotope labelling by amino acids in cell culture [SILAC]-hFXN [S^81^GTLGHPGSLDETTYER^97^] in which the [^12^C_6_]-lysine and [^12^C_6_]-leucine were replaced with [^13^C_6_^15^N_1_]-lysine and [^13^C_6_]-leucine), to tissue samples before the analysis (Fig. 2). Mature SILAC-hFXN (81–210) is 98.5% homologous and 92.3% identical to the predicted sequence of mature mFXN (78–207). The hFXN protein standard was prepared with a 6 × His tag and the SILAC-hFXN internal standard prepared with a 6 × His tag as previously described^30,32^.

### Tissue lysis.

Mouse heart and liver tissue samples were cut into small pieces while still frozen, and 7–100 mg pieces were transferred to LoBind Eppendorf tubes containing 1 mL RIPA lysis buffer (supplemented with 1 mM EDTA, 1 × complete protease cocktail, 1 mM DTT). Tissue homogenization was conducted on ice using a sonication probe (30 pulses at setting 5). Lysed samples were centrifuged at 17,000 × *g* at 4°C for 10 min and the supernatant transferred to a separate tube. For heart samples, 50 ng of SILAC-hFXN standard was added and the entire sample was analyzed. For liver samples, 500 μL of the homogenate (~ 50%) was withdrawn, and 50 ng of SILAC-hFXN standard was added (i.e., only half of the sample was analyzed). Sample volumes were adjusted to standardize tissue amounts across samples.

### mAb-bead cross-linking.

Anti-FXN recombinant rabbit mAb (Abcam; Cambridge, UK; cat. # ab21914) was cross-linked to magnetic protein G beads using DMP^30^ (Fig. 2). This made it possible to thoroughly wash the beads to remove all interfering matrix contaminants before eluting the mature FXN proteoforms for 2D-nano-UHPLC-PRM/HRMS analysis, unlike previous IP studies^31^. Analyses could then be conducted without contamination of the mass spectrometer’s ion source or degradation of the LC column. Protein G magnetic Dynabeads (5 mg; 165 μL; ThermoFisher Scientific; Waltham, MA; cat. # 10009D) were washed three times with PBS containing 0.02% Tween (buffer A) and incubated with 40 μg (40 μL in 460 μL PBS) of a recombinant rabbit mAb against the FXN protein (cross-reactive with both mFXN and hFXN) on a rotator at 4°C overnight. The mAb-bound protein G beads were washed twice with 1 mL cross-linking buffer (0.2 M triethanolamine, pH 8.0) and incubated with 13 mg DMP in 2 mL cross-linking buffer on a rotator at room temperature for 1 h. The mAb-crosslinked G beads were quenched with 2 mL blocking solution (0.1 ethanolamine, pH 8.5) for 1 h at room temperature. The beads were washed with buffer A three times and stored in 1 mL of buffer A at 4°C until used (Fig. 2).

### FXN IP.

FXN protein IP was performed following a previously described protocol with minor modifications^31^. Briefly, a portion of tissue homogenate (typically 500 μL) was mixed with 500 μL ice-cold RIPA lysis buffer (supplemented with 1 × complete protease cocktail,1 mM DTT). Mature SILAC-hFXN (50 ng) containing [^13^C_6_]-Leucine was spiked into each sample to serve as the internal standard. Each sample was transferred to a 2 mL LoBind Eppendorf tube containing 100 μL G beads cross-linked to an FXN mAb (0.5 mg) and incubated on a rotator at 4°C overnight. The next day, the supernatant was removed, and the cross-linked beads were washed two times with 1 mL buffer A. The beads were then transferred to a LoBind Eppendorf tube and washed with 1 mL PBS. Next, 100 μL elution buffer (300 mM acetic acid and 10% acetonitrile) was added to the beads, which were eluted at 1,000 rpm at 37°C for 1 h. The elution buffer was then transferred to another 1.5 mL LoBind Eppendorf and dried under nitrogen flow. Dried samples were then dissolved in 50 μL 25 mM aqueous NH_4_HCO_3_ solution containing 500 ng trypsin protease and digestion was performed at 37°C overnight prior to 2D-nano-UHPLC-PRM/HRMS analysis. Calibration standards were prepared by spiking 1, 2, 15, 30, 50, 600 and 1500 ng of mature hFXN into a solution of 50 ng of mature SILAC-hFXN in 5% bovine serum albumin (BSA), and analysis of the calibration standards was performed alongside the samples following the same protocol.

### Standard curves.

Linear standard curves were obtained for the FXN peptide S^81^GTLGHPGSLDETTYER^97^) used to calculate mFXN and hFXN levels over a 1–50 ng range as previously described^33^ (a representative standard curve shown in Supplementary Fig. S1a). Different standard curves were used to quantify mFXN and hFXN > 50 ng, as these quantities distorted the lower end of the curve (Supplementary Fig. S1b). Transitions that were used to monitor the peptides are shown in Supplementary Table S1. Back-calculated values for the authentic standards used to prepare the standard curves were within acceptable limits (Supplementary Table S2).

### 2D-Nano-UHPLC-PRM/HRMS.

Analyses were conducted using a high-resolution Q-Exactive HF hybrid quadrupole-orbitrap mass spectrometer coupled to a Dionex Ultimate 3000 RSLCnano with capillary flowmeter chromatographic systems (ThermoFisher Scientific; San Jose, CA) as previously described with minor modifications (Fig. 2)^1^. The 2D system was set up in a pre-concentration mode composed of a ten-port valve, one nanopump for delivering solvents to analytical columns, and a micropump for delivering solvents to trapping columns. The 2D-nano-UHPLC-PRM/HRMS system was controlled by Xcalibur software (version 4.3) from the Q-Exactive mass spectrometer. The UHPLC trapping column was an Acclaim PepMap C18 cartridge (0.3 mm × 5 mm, 100 Å; ThermoFisher Scientific; San Jose, CA) and the analytical column was a C18 AQ nano-UHPLC column with a 10 μm pulled tip (75 μm × 25 cm, 3 μm particle size; Columntip; New Haven, CT). Samples (8 μL) were injected using the microliter-pickup injection mode. Loading solvent was water:acetonitrile (99.7:0.3 v:v) containing 0.2% formic acid. During sample loading, the valve stayed in the loading position (1–2) and solvent was loaded at 10 μL/min for 3 min. During the elution and analysis steps, the valve stayed in the injection position (1–10), the trapping column was connected to the nanopump and the analytical column, and samples were back-flushed into the analytical column. Washing of the trapping column using the nanopump continued until 5 min before the end of the run. Samples were eluted in the mass spectrometer with a linear gradient at a flow rate of 0.4 μL/min. Solvent A was water:acetonitrile (99.5:0.5 v:v) containing 0.1% formic acid, and solvent B was acetonitrile:water (98:2 v:v) containing 0.1% formic acid. The gradient on the analytical column was as follows: 2% B at the start, 5% B at 10 min, 55% B at 45 min, 98% B at 60 min, held for 15 min, then re-equilibrated at 2% B from 70−80 min. Ionization was conducted using a Nanospray Flex ion source (ThermoFisher Scientific; San Jose, CA). Mass spectrometer operating conditions were as follows: spray voltage = 3500 V; ion transfer capillary temperature = 275°C; ion polarity = positive; S-lens radiofrequency (RF) level = 55; and in-source collision-induced dissociation (CID) = 1.0 eV. The PRM/HRMS parameters were as follows: resolution = 60,000; automatic gain control (AGC) target = 2e5; maximum ion trap (IT) = 80 ms; loop count = 5; isolation window = 2.0 Da; and normalized collision energy (NCE) = 25 (Supplementary Table S1).

### Data analyses.

Protein quantification was performed using Skyline software (version 23.1; MacCoss Laboratory, University of Washington; Seattle, WA). ^34^ The peak area ratio of each PRM/HRMS transition for each unlabeled/light (L) peptide to labeled/heavy (H) peptide was calculated by Skyline software and used for absolute quantification. The most intense PRM/HRMS transition of [M + 3H]^3+^ (m/z 611.3002) to y^4+^ (568.2726) transition for S^81^GTLGHPGSLDETTYER^97^ (L = [^13^C_6_]-leucine) was used for the internal standard. The most intense PRM/HRMS transition of [M + 2H]^2+^ (m/z 940.9473) to y^7+^ (m/z 883.3792) for L^78^GTLDNPSSLDETAYER^94^ was used to quantify mature mFXN. The most intense PRM/HRMS transition of [M + 3H]^3+^ (m/z 607.2867) to y^4+^ (m/z 568.2726 for S^81^GTLGHPGSLDETTYER^97^ was used to quantify mature hFXN. Concentrations of mature mFXN and hFXN were determined from the L/H ratio of each N-terminal peptide to S^81^GTLGHPGSLDETTYER^97^ and interpolation from the standard curve.

### Ethics declarations.

All animal protocols and surgical experiments on mice were performed at the Belfer Gene Therapy Core Facility in accordance with and approved by the Institutional Animal Care and Use Committee (IACUC) of Weill Cornell Medicine.

## RESULTS

### Effect of hFXN gene therapy on mature mFXN levels in mouse heart tissue

**Figure 3:**
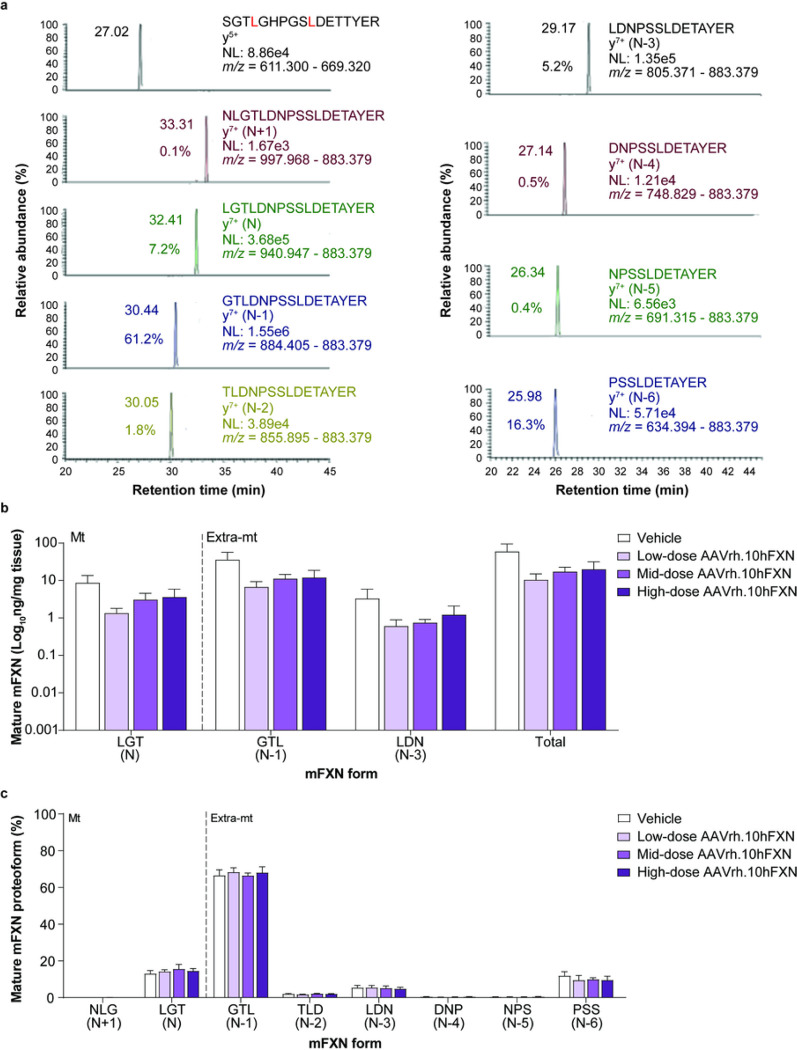
Detection and quantification of mature mFXN and its N-terminal tryptic peptides in the mouse heart following hFXN gene therapy. (**a**) Representative chromatograms from 2D-nano-UHPLC-PRM/HRMS analysis of mature mFXN N-terminal tryptic peptides in the heart of a mouse administered a high dose (1.8e13 gc/kg) of AAVrh.10hFXN. y-ion indicates the number of amino acids from the carboxy terminus that are present in this ion. Peptide retention times and relative amounts are shown on the left of the relevant signal. (**b**) Absolute concentrations of Mt and Extra-mt mature mFXN proteoforms in mouse heart after administration of one of three different doses of AAVrh.10hFXN (low dose = 1.8e12 gc/kg, n = 6; mid dose = 5.7e12 gc/kg, n = 4; high dose = 1.8e13 gc/kg, n = 8) or vehicle (n = 5). While there were six mice treated with mid-dose AAVrh.10hFXN, insufficient heart sample precluded analysis of two mice. (**c**) Relative amounts of mature mFXN proteoforms in mice after administration of the same doses of AAVrh.10hFXN or vehicle (sample sizes as above). (**b,c**) Data are expressed as mean ± s.e.m. 2D-nano-UHPLC-PRM/HRMS = two-dimensional nano-ultra-high performance liquid chromatography-parallel reaction monitoring high-resolution mass spectrometry; AAVrh.10hFXN = adeno-associated virus rhesus serotype 10 encoding human frataxin; Extra-mt = extra-mitochondrial; hFXN = human frataxin; mFXN = mouse frataxin; Mt = mitochondrial; m/z = mass-to-charge ratio; NL = normalized signal level; s.e.m. = standard error of the mean.

2D-nano-UHPLC-PRM/HRMS was used to detect mature mFXN and its truncated forms in the mouse heart following hFXN gene therapy. A representative chromatogram of mature mFXN in the mouse heart is shown in Fig. 3a. There was robust detection of the N-terminal peptide (SGT**L**GHPGS**L**DETTYER) of the mature form of the internal standard (SILAC-hFXN), the mature mFXN N-terminal peptide (LGTLDNPSSLDETAYER [denoted N or LGT]), and the N-terminally truncated mFXN proteoforms (denoted N minus 1 to N minus 6 [N-1 to N-6], or by their N-terminal triplet amino acid sequence, i.e., GTL for N-1, TLD for N-2, and so on). Further, only product ions from MH_3_^3+^ from the internal standard N-terminal tryptic-peptide S^81^GT**L**GHPGS**L**DETTYER^97^ were detected (Fig. 3a), excluding the possibility that trypsin-independent proteolysis during the analytical procedure leads to the formation of the truncated FXN proteoforms observed in this study. In contrast, product ions from MH_2_^2+^ (including the intense y_7_^+^-ion) of the predicted (N-1)-mFXN tryptic-peptide G^79^TLDNPSSLDETAYER^94^ were present in mouse heart, together with relatively weak product ions derived from three other major truncated forms (N-2, N-3, and N-6; Fig. 3a). These findings are very similar to those reported for mice that did not undergo gene therapy, indicating that gene therapy did not alter endogenous mFXN biosynthesis and processing in the heart^31^.

Levels of mature mFXN were next measured in the mouse heart following administration of vehicle or low-dose (1.8e12 gc/kg), mid-dose (5.7e12 gc/kg), or high-dose (1.8e13 gc/kg) AAVrh.10hFXN. 2D-nano-UHPLC-PRM/HRMS ion transitions used for the analysis of mature FXN forms are shown in Supplementary Table S1. Mean levels of total mature mFXN in heart tissue of mice receiving one of three AAVrh.10hFXN doses ranged from a low of 1.3 to a high of 193.5 ng/mg tissue with an average of 25.6 ± 9.0 ng/mg (mean ± s.e.m.; n = 23; median = 7.7 ng/mg) and showed no clear dose-dependence (Fig. 3b and Supplementary Table S3). The relative abundance of the N peptide (compared with truncated mFXN forms) remained remarkably constant in the heart (14.5%; Fig. 3c and Supplementary Table S3), with levels approximating those reported in mice not subjected to gene therapy in a previous study^31^. The N-1 proteoform was the most abundant and represented 67.6% of total mature mFXN (Fig. 3c and Supplementary Table S3). The mFXN tryptic peptide N^77^LGTLDNPSSLDETAYER^97^, denoted N + 1, was monitored to determine whether full length mFXN or elongated forms (compared to mature mFXN) resulting from inhibition of MPP^28,29^ were present in mouse hearts. The N + 1 peptide represented an overall mean of only 0.1% of total mFXN in the mouse heart (n = 23) (Fig. 3a and Supplementary Table S3). This indicated near-complete processing of full length/elongated mFXN into mature/truncated mFXN proteoforms.

### Effect of hFXN gene therapy on mature mFXN levels in mouse liver tissue

**Figure 4:**
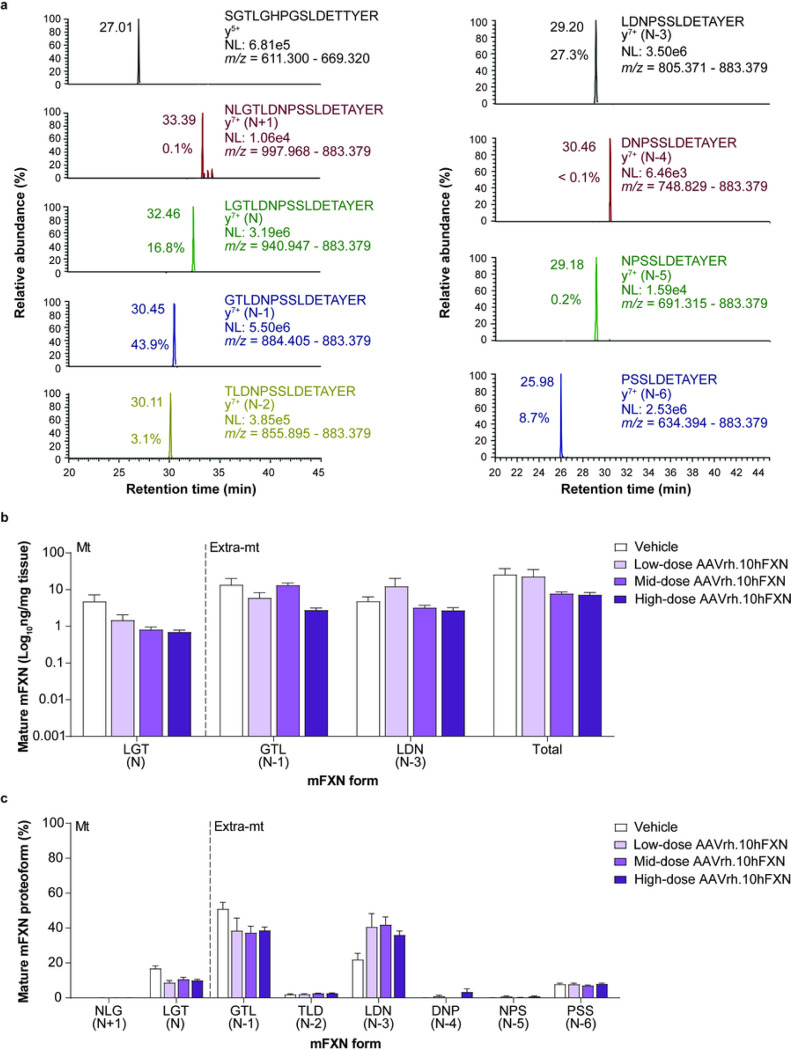
Detection and quantification of mature mFXN and its N-terminal tryptic peptides in the mouse liver following hFXN gene therapy. (**a**) Representative chromatograms from 2D-nano-UHPLC-PRM/HRMS analysis of mature mFXN N-terminal tryptic peptides in the liver of a mouse administered a high dose (1.8e13 gc/kg) of AAVrh.10hFXN. y-ion indicates the number of amino acids from the carboxy terminus that are present in this ion. Peptide retention times and relative amounts are shown on the left of the relevant signal. (**b**) Absolute concentrations of Mt and Extra-mt mature mFXN proteoforms in mouse liver after administration of one of three different doses of AAVrh.10hFXN (low-dose = 1.8e12 gc/kg, n = 6; mid-dose = 5.7e12 gc/kg, n = 6; high-dose = 1.8e13 gc/kg, n = 8) or vehicle (n = 5). (**c**) Relative amounts of mature mFXN in mice after administration of the same doses of AAVrh.10hFXN or vehicle (sample sizes as above). (**b,c**) Data are expressed as mean ± s.e.m. AAVrh.10hFXN = adeno-associated virus rhesus serotype 10 encoding human frataxin; 2D-nano-UHPLC-PRM/HRMS = two-dimensional nano-ultra-high performance liquid chromatography-parallel reaction monitoring high-resolution mass spectrometry; AAVrh.10hFXN = adeno-associated virus rhesus serotype 10 encoding human frataxin; Extra-mt = extra-mitochondrial; hFXN = human frataxin; mFXN = mouse frataxin; Mt = mitochondrial; m/z = mass-to-charge ratio; NL = normalized signal level; s.e.m. = standard error of the mean.

A representative 2D-nano-UHPLS-PRM/HRMS chromatogram of mature mFXN in the mouse liver is shown in Fig. 4a. Similar to heart tissue findings, in mouse liver there was robust detection of the mature SILAC-hFXN internal standard N-terminal peptide (SGT**L**GHPGS**L**DETTYER), the mFXN N peptide (LGTDNPSSLDETAYER), and the N-terminally truncated forms (N-1 to N-6). These results indicate that synthesis and processing of endogenous mFXN in the liver is not altered following AAVrh.10hFXN gene therapy, as similar findings were observed in mice that did not undergo gene therapy^31^. Levels of mature mFXN were measured in the mouse liver following administration of vehicle or low-dose (1.8e12 gc/kg), mid-dose (5.7e12 gc/kg), or high-dose (1.8e13 gc/kg) AAVrh.10hFXN. Mean levels of total mature mFXN in mouse liver administered vehicle or one of the three AAVrh.10hFXN doses ranged from a low of 2.7 to a high of 81.7 ng/mg tissue, or an average of 14.9 ± 3.9 ng/mg (mean ± s.e.m.; n = 25; median = 9.5 ng/mg) and showed no dose-dependence (Fig. 4b and Supplementary Table S4). The relative abundance of the N form (compared with truncated mFXN forms) in mouse liver was 11.3% (Fig. 4c and Supplementary Table S4), and similar to mouse heart findings, approximated N-proteoform levels found in mice not subjected to gene therapy in a previous study^31^. The extra-mitochondrial N-1 form was the most abundant mFXN proteoform and represented 40.8% of total mature mFXN (Fig. 4c). Consistent with previous studies^31^, a substantial amount of the N-3 truncated form (35.8%) was detected in the mouse liver. The N + 1 peptide (monitored to determine the presence of elongated forms of mFXN) represented an overall mean of only 0.1% of total mFXN in the mouse liver (n = 25), indicating near-complete processing of full length/elongated mFXN into mature/truncated mFXN proteoforms (Fig. 4a and Supplementary Table S4).

### Mature hFXN expression in mouse heart tissue following hFXN gene therapy

**Figure 5:**
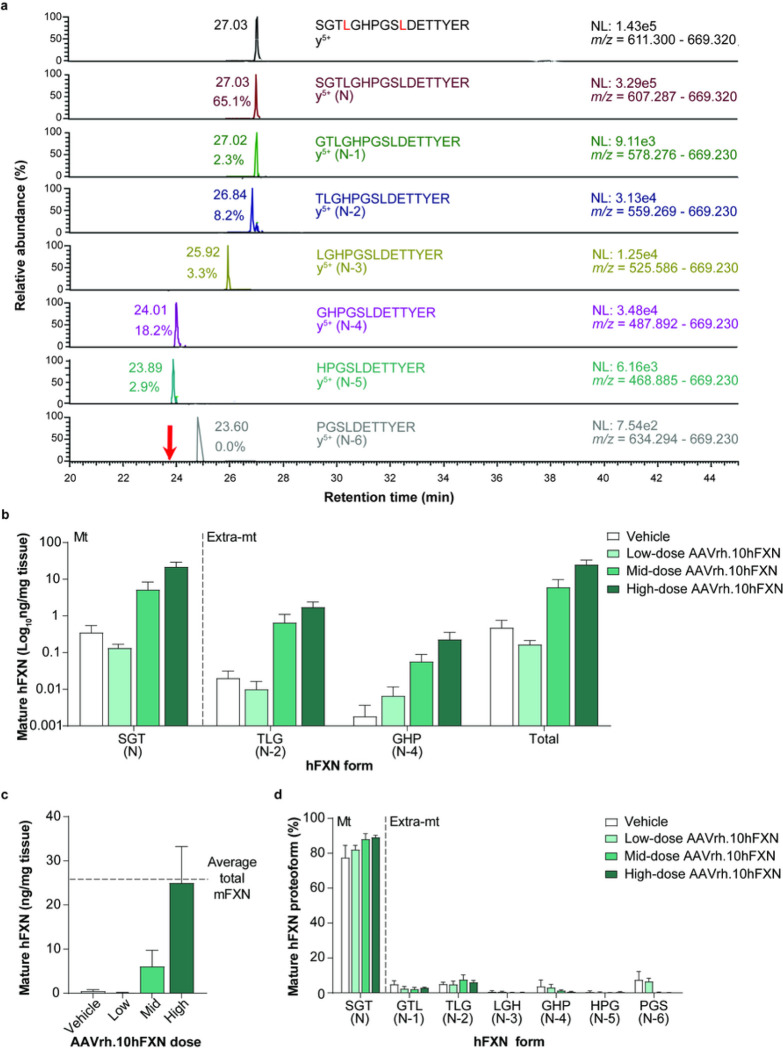
Detection and quantification of mature hFXN and its N-terminal tryptic peptides in the mouse heart following hFXN gene therapy. (**a**) Representative chromatograms from 2D-nano-UHPLC-PRM/HRMS analysis of mature hFXN N-terminal tryptic peptides in the heart of a mouse administered a high dose (1.8e13 gc/kg) of AAVrh.10hFXN. The red arrow indicates the retention time of the peptide. y-ion indicates the number of amino acids from the carboxy terminus that are present in this ion. Peptide retention times and relative amounts are shown on the left of the relevant signal. (**b**) Absolute concentrations of Mt and Extra-mt mature hFXN proteoforms in mouse heart after administration of one of three different doses of AAVrh.10hFXN (low dose = 1.8e12 gc/kg, n = 6; mid dose = 5.7e12 gc/kg, n = 4; high dose = 1.8e13 gc/kg, n = 8) or vehicle (n = 5). While there were six mice treated with mid-dose AAVrh.10hFXN, insufficient heart sample precluded analysis of two mice. (**c**) Dose-dependent increase in total mature hFXN levels in mouse heart after administration of the same doses of AAVrh.10hFXN or vehicle (sample sizes as above). While there were six mice treated with mid-doseAAVrh.10hFXN, insufficient heart sample precluded analysis of two mice. The dotted line represents endogenous total mature mFXN levels (25.6 ng/mg). (**d**) Relative amounts of mature hFXN proteoforms in mouse heart after administration of the same doses of AAVrh.10hFXN or vehicle (sample sizes as above). (**b-d**) Data are expressed as mean ± s.e.m. 2D-nano-UHPLC-PRM/HRMS = two-dimensional nano-ultra-high performance liquid chromatography-parallel reaction monitoring high-resolution mass spectrometry; AAVrh.10hFXN = adeno-associated virus rhesus serotype 10 encoding human frataxin; Extra-mt = extra-mitochondrial; hFXN = human frataxin; mFXN = mouse frataxin; Mt = mitochondrial; m/z = mass-to-charge ratio; NL = normalized signal level; s.e.m. = standard error of the mean.

2D-nano-UHPLC-PRM/HRMS was used to detect mature hFXN following hFXN gene therapy and assess whether it underwent truncation similar to mFXN in the mouse heart. A representative chromatogram of mature hFXN in mouse heart tissue is shown in Fig. 5a. There was a robust detection of both N-terminal peptides representing mature SILAC-hFXN internal standard and mature hFXN. In addition, N-terminal truncations of hFXN were detected, which corresponded to the N-1 (GTL), N-2 (TLG), N-3 (LGH), and N-6 (PGS) extra-mitochondrial forms (Fig. 5a).

Mature hFXN was detected in all heart samples from mice treated with AAVrh.10hFXN (Fig. 5b and Supplementary Table S5). Levels of hFXN increased in a dose-dependent manner, with mean total mature hFXN in mouse heart tissue following administration of low-, mid-, or high-dose AAVrh.10hFXN (0.2, 6.1, and 25.0 ng/mg, respectively; Fig. 5c). At the highest dose, the mean total mature hFXN level was similar to the mean level of endogenous total mature mFXN (Fig. 5c). The mitochondrial N peptide represented 84.6% of total mature hFXN levels in the mouse heart (Fig. 5d and Supplementary Table S5). Of the 15.4% of mature hFXN that underwent truncation, a mean of 3.1% corresponded to the N-1 form and a mean of 5.8% corresponded to the N-2 form (Fig. 5d and Supplementary Table S5).

### Mature hFXN expression in mouse liver tissue following hFXN gene therapy

**Figure 6:**
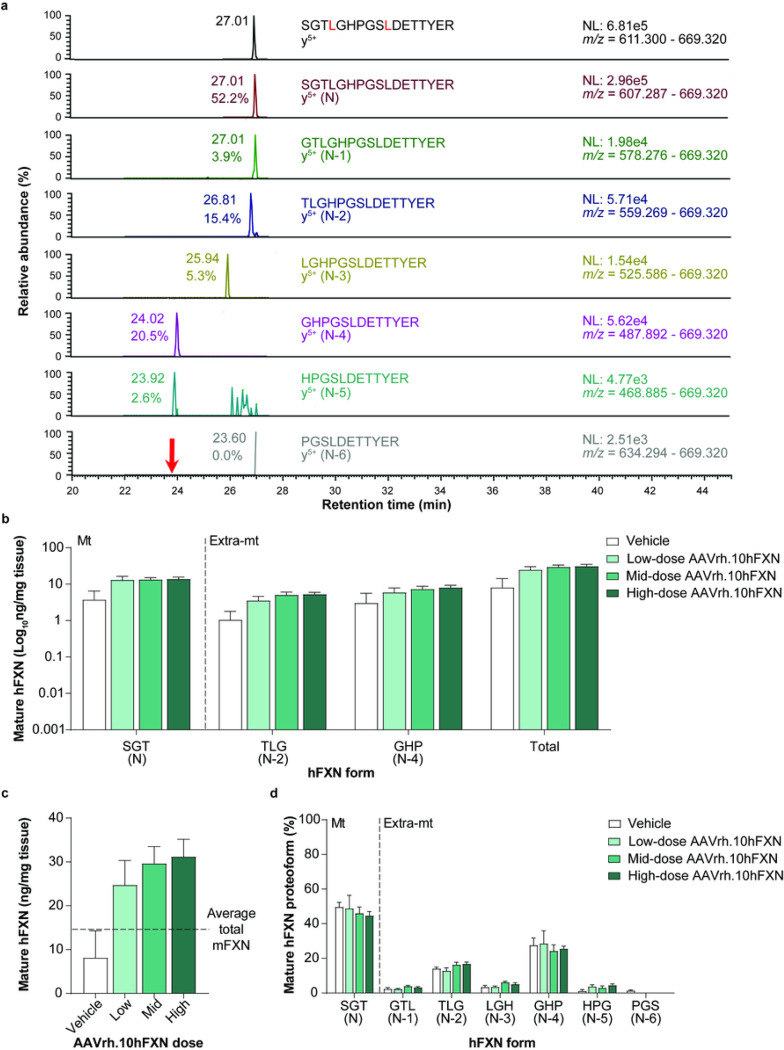
Detection and quantification of mature hFXN and its N-terminal tryptic peptides in the mouse liver following hFXN gene therapy. (**a**) Representative chromatograms from 2D-nano-UHPLC-PRM/HRMS analysis of mature hFXN N-terminal tryptic peptides in the liver of a mouse administered a high dose (1.8e13 gc/kg) of AAVrh.10hFXN. The red arrow indicates the retention time of the peptide. y-ion indicates the number of amino acids from the carboxy terminus that are present in this ion. Peptide retention times and relative amounts are shown on the left of the relevant signal. (**b**) Absolute concentrations of Mt and Extra-mt mature hFXN in mouse liver after administration of one of three different doses of AAVrh.10hFXN (low-dose = 1.8e12 gc/kg, n = 6; mid-dose = 5.7e12 gc/kg, n = 6; high-dose = 1.8e13 gc/kg, n = 8) or vehicle (n = 5). (**c**) Dose-dependent increase in total mature hFXN levels in mouse liver after administration of the same doses of AAVrh.10hFXN or vehicle (sample sizes as above). The dotted line represents endogenous total mature mFXN levels (14.9 ng/mg). (**d**) Relative amounts of mature hFXN proteoforms in mouse liver after administration of the same doses of AAVrh.10hFXN or vehicle (sample sizes as above). (**b-d**) Data are expressed as mean ± s.e.m. 2D-nano-UHPLC-PRM/HRMS = two-dimensional nano-ultra-high performance liquid chromatography-parallel reaction monitoring high-resolution mass spectrometry; AAVrh.10hFXN = adeno-associated virus rhesus serotype 10 encoding human frataxin; Extra-mt = extra-mitochondrial; hFXN = human frataxin; mFXN = mouse frataxin; Mt = mitochondrial; m/z = mass-to-charge ratio; NL = normalized signal level; s.e.m. = standard error of the mean.

A representative 2D-nano-UHPLC-PRM/HRMS chromatogram of mature hFXN in mouse liver is shown in Fig. 6a. Similar to heart tissue findings, there was a robust detection of mature hFXN N peptide in the livers of mice treated with low-, mid-, or high-dose AAVrh.10hFXN (Fig. 6b and Supplementary Table S6). Total mature hFXN levels increased in a dose-dependent manner (Fig. 6c), with higher heart hFXN levels being associated with higher hFXN liver levels (Figs. 5c and 6c). Mean levels of total mature hFXN in mouse liver tissue following administration of low-, mid-, or high-dose AAVrh.10hFXN were 24.7, 29.6, and 31.2 ng/mg, respectively (Fig. 6c).

Mature hFXN expressed in mouse liver tissue was most frequently identified in N-2 (15.2%) and N-4 (26.4%) forms (Fig. 6d), which contrasts with the relatively lower abundance of N-2 form found in the heart (5.8%; Fig. 5d). The relative amount of the hFXN N form (compared with truncated hFXN forms) was lower in liver (47.0%) versus heart tissues (84.6%; Figs. 5d, 6d, and Supplementary Tables S5 and S6).

## DISCUSSION

Several studies have shown the potential for AAV-based gene therapy to rescue the cardiac manifestations of FRDA^25,36,37^, but its success has thus far been limited by difficulties with achieving and maintaining hFXN levels that are sufficiently efficacious but low enough to avoid cardiac toxicity and subsequent decline of efficacy^25,26^. Given these challenges, novel gene replacement approaches for FRDA need to first undergo comprehensive analysis in animal models to test and optimize efficacy and to minimize toxicity prior to evaluating the therapeutic potential in patients.

The aim of the present pre-clinical study was to evaluate hFXN expression following hFXN gene therapy in mice. The primary objective was to determine whether mature hFXN could be expressed at levels close to endogenous mature mFXN^31^ after administration of recombinant AAVrh.10 encoding hFXN (AAVrh.10hFXN). In addition, this study aimed to determine whether hFXN expressed in mouse tissues underwent truncation (similar to mFXN) ^31^, a process that does not appear to occur to hFXN in human tissues^31^.

Findings from this study demonstrate that IV administration of AAVrh.10hFXN gene therapy leads to dose-dependent cardiac and hepatic expression of mature hFXN in mice (Figs. 5d and 6d), and that hFXN undergoes processing (similar to endogenous mFXN) to produce truncated mature forms of hFXN that have not been observed in human tissues^31^. Unlike mFXN, which is primarily truncated to an N-1 form, AAVrh.10hFXN-delivered hFXN is primarily truncated to an N-2 form in mouse hearts (Fig. 5b). Though the impact of this differential processing of hFXN versus mFXN is unclear, at the doses administered in this study, AAVrh.10hFXN-derived total hFXN protein does not appear to be expressed at levels exceeding the average total endogenous mFXN level in mouse heart tissue (Fig. 5c), as has been previously reported in animal models^25,26,38^.

Cytosolic full-length hFXN rapidly translocate to the mitochondria where it undergoes two sequential cleavages by MPP to produce mitochondrial mature hFXN containing the N-terminal tryptic peptide (S^81^GTLGHPGSLDETTYER^97^). Mature hFXN has never been detected outside of the mitochondria in humans^39^. Therefore, it is reasonable to surmise that when full-length hFXN is expressed in the cytosol of mouse cells, it will similarly translocate to the mitochondria and undergo sequential MPP-mediated cleavages on the N-terminal side of leucine-42 and serine-81 to give rise to mature hFXN (Fig. 1) ^28,29^. This mature hFXN form was the major form (80–90%) observed in the mouse heart following hFXN gene therapy (Fig. 3b). The lack of significant amounts of mature (N-1)-hFXN found in the mouse heart may be attributed to the amino-terminal sequence differences. Mature mFXN has an N-terminal leucine (Fig. 1), which violates the N-terminal end rule and is thus predicted to be metabolically unstable^40^. In contrast, mature hFXN has a metabolically stable serine residue at its N-terminus (Fig. 1). At higher doses of AAVrh.10hFXN, mature (N-2)-hFXN accounted for approximately 6% of the total hFXN detected in the mouse heart, which was similar to the level of mature (N-1)-mFXN detected in the mouse heart (Supplementary Table S5). This suggests that the protease reasonable for the cleavage of mature mFXN on the N-terminal side of glycine-79 might be responsible for cleavage of mature hFXN on the N-terminal side of threonine-83 (Fig. 1). Unlike the extra-mitochondrial location of the mature (N-1)-mFXN peptide, the sub-cellular localization of other truncated forms of mFXN in the mouse heart have not yet been delineated.

Compared with mouse heart, more abundant truncation of hFXN was observed in mouse liver, such that mature (N)-hFXN only accounted for 47.0% in the liver (Supplementary Table S6). Mature (N-2)- and (N-4)-hFXN were the other two major FXN proteoforms, accounting for 15.2% and 26.4% of the total mature hFXN, respectively. As was observed in mouse heart tissue, the enzyme responsible for generating mature (N-1)-mFXN in mouse liver generated very little mature (N-1)-hFXN (Supplementary Table S6). It is curious that mature (N-6)-hFXN was not detected in mouse liver (Supplementary Table S6), whereas larger amounts of cardiac and hepatic (N-6)-mFXN were detected (Supplementary Tables S3 and S4). It is conceivable that the protease responsible for generating mature (N-3)-mFXN in the liver (Supplementary Table S4) was also able to generate mature (N-4)-hFXN (Supplementary Table S6). However, the sub-cellular localization of these truncated forms of mFXN and hFXN in mouse liver remains unknown.

In contrast to the enzyme-linked immunosorbent assays (ELISAs) typically used for FXN detection, the 2D-nano-UHPLC-PRM/HRMS method can distinguish between mFXN and hFXN proteins within the same sample in a single assay. Unlike full-length mFXN, which is primarily processed to N and N-1 forms, full-length hFXN is primarily processed to N and N-2 forms in AAVrh.10hFXN-treated mouse hearts. The effect of this differential processing on treated mice is unclear, however the hFXN levels achieved in mouse heart were not highly overexpressed as has been previously reported to cause toxicity (Fig. 3c) ^25^. Furthermore, in other studies, administration of similar doses of AAVrh.10hFXN to mild or severe mouse models of FRDA improved the cardiac disease-relevant phenotypes without inducing the type of toxicity generally observed at the highest dose^37,41^.

In summary, study results showed that IV administration of AAVrh.10hFXN at doses known to be effective in severe FRDA mouse models results in cardiac and hepatic mature hFXN levels that approximate endogenous mFXN levels in WT mice. The application of AAVrh.10hFXN gene therapy in patients with FRDA is currently being tested to examine the potential of delivering safe and therapeutic hFXN levels.
